# Gut microbiome and kidney disease: a bidirectional relationship

**DOI:** 10.1007/s00467-016-3392-7

**Published:** 2016-04-29

**Authors:** Souhaila Al Khodor, Ibrahim F. Shatat

**Affiliations:** 1 Infectious Disease Unit, Division of Translational Medicine, Sidra Medical and Research Center, PO Box 26999, Doha, Qatar; 2 Pediatric Nephrology and Hypertension, SIDRA Medical and Research Center, Doha, Qatar; 3 Medical University of South Carolina, Charleston, SC USA; 4 Weill Cornell Medical College, New York, NY USA

**Keywords:** Hypertension, Dysbiosis, Microbiota, Prebiotics, Probiotics, End-stage renal disease, Chronic kidney disease, Hemodialysis, Peritoneal dialysis, Acute kidney injury, Transplantation

## Abstract

Recent technological advances and efforts, including powerful metagenomic and metatranscriptomic analyses, have led to a tremendous growth in our understanding of microbial communities. Changes in microbial abundance or composition of human microbial communities impact human health or disease state. However, explorations into the mechanisms underlying host–microbe interactions in health and disease are still in their infancy. Although changes in the gut microbiota have been described in patients with kidney disease, the relationships between pathogenesis, mechanisms of disease progression, and the gut microbiome are still evolving. Here, we review changes in the host–microbiome symbiotic relationship in an attempt to explore the bidirectional relationship in which alterations in the microbiome affect kidney disease progression and how kidney disease may disrupt a balanced microbiome. We also discuss potential targeted interventions that may help re-establish this symbiosis and propose more effective ways to deploy traditional treatments in patients with kidney disease.

## Background

The human body harbors a complex community of bacteria, archaea, viruses, and eukaryotic microbes that inhabit interactive interfaces exposed or connected to the external environment [1]. This collection of microorganisms consists of about 100 trillion microbial cells called the human microbiota, and the genes encoded by these microbes collectively form the human microbiome [2]. The microbiome is an integral part of the human genetic landscape; therefore, for a complete understanding of our genetics, it is essential to study the composition of our microbiome [2].

**Fig. 1:**
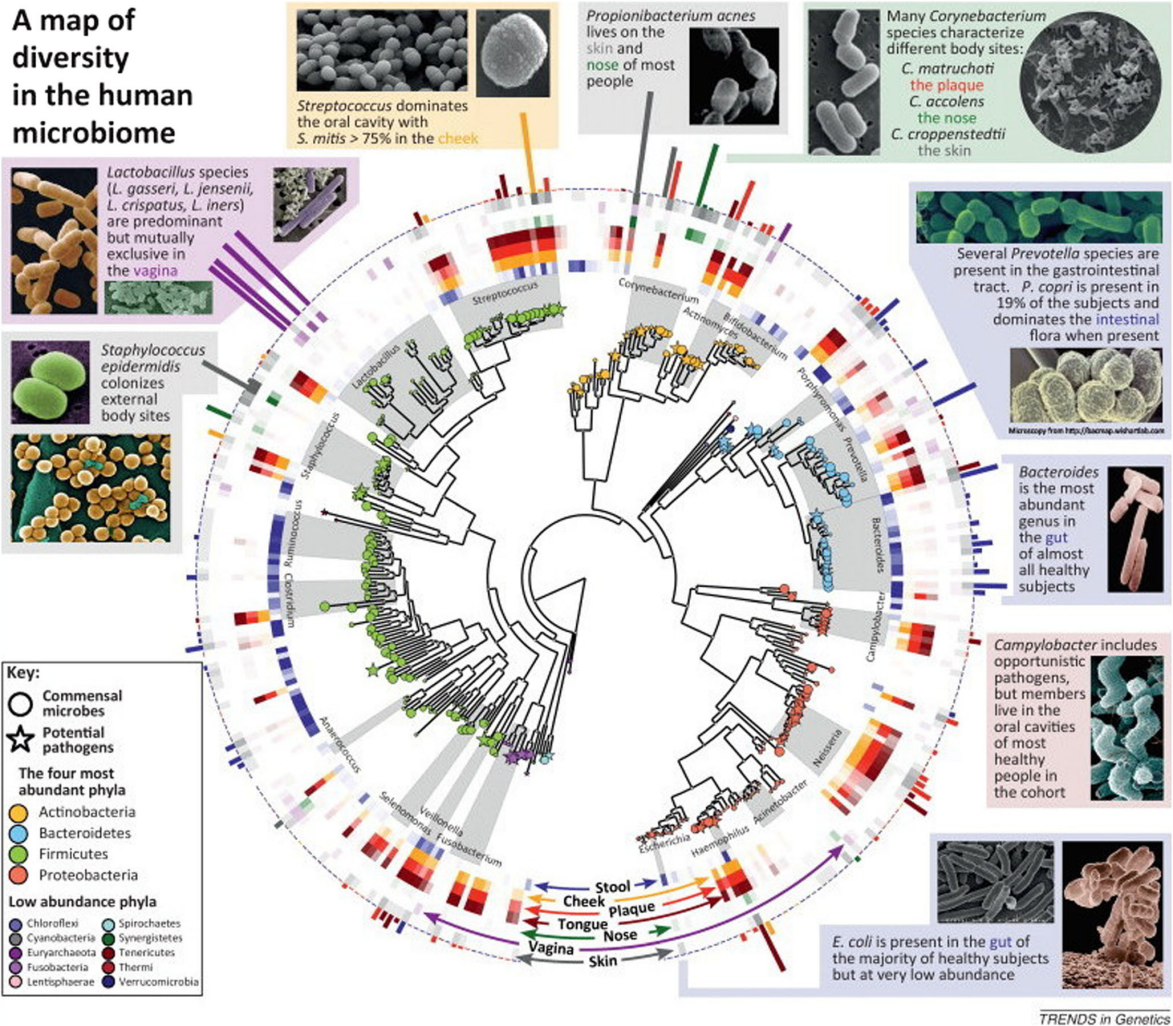
Diversity in the human microbiome. The human microbiome is dominated by four phyla: Actinobacteria, Bacteroidetes, Firmicutes, and Proteobacteria. In the center is a phylogenetic tree of organisms abundant in the human microbiome. Commensal microbes are indicated by *circles*, and potential pathogens are indicated by *stars*. The *middle ring* corresponds to body sites at which various taxa are abundant and is color-coded by site [e.g., *Ruminococcus* (*blue*) is found mostly in the gut, whereas *Lactobacillus* (*purple*) is found mostly in the vagina]. *Bar heights* on the outside of the circle are proportional to taxa abundance at the body site of greatest prevalence [e.g., *Streptococcus mitis* (*yellow*) dominates the inside of the cheek, whereas the gut is abundant in a variety of *Bacteroides*]. The *intensity of external colors* corresponds to species prevalence in each body site (adapted with permission from [12])

Microbes have a tremendous impact on human health and well-being, with the potential to impact our physiology both in health and in disease [3]. They protect against invading pathogens, educate our immune cells, contribute to various metabolic functions, and—through these basic functions—affect directly or indirectly most of our physiologic functions [4, 5]. The breakthroughs in high-throughput techniques used to analyze the composition of the microbiome have substantially advanced our knowledge of the microbial communities colonizing various human niches [6]. Moreover, the decrease in cost of sequencing using those high-throughput technologies has enabled large-scale studies of the human microbiome [7–9]. Large-scale studies revealed that each body site, such as the oronasopharyngeal sphere, skin, vagina, and gastrointestinal tract, contains ecological communities of microbial species that exist in a mutualistic relationship with the host, also known as symbiosis [1]. Each person’s microbiome is thought to be unique [10]. Differences in species, abundance, and diversity of microbial communities within the same individual and among different individuals and various body sites have been previously described [10, 11] and are summarized in Fig. 1 [12]. Much of this intrapersonal diversity has been attributed to differences in host genetics, geographical origin and location, age, lifestyle, eating habits, and early microbial exposure, as well as antibiotics or probiotics intake [10]. Early in life, the mode of birth delivery, gestational age, hospitalization, diet, and the nature of feeding are among the factors that contribute to microbiome diversity [13].

### Methodologies used to study the microbiome

**Fig. 2:**
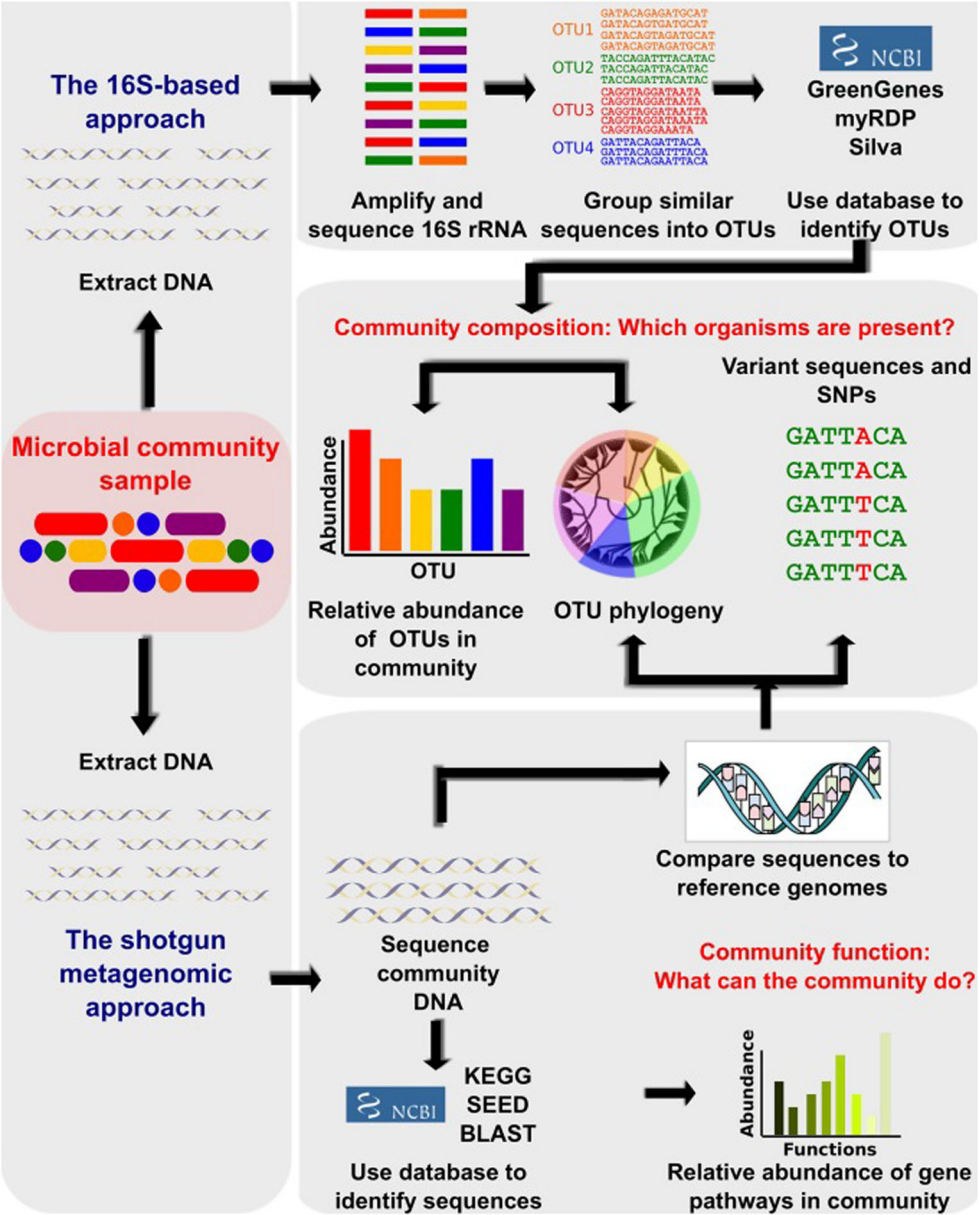
Bioinformatic methods for functional metagenomics. Microbial community samples typically contain many species of bacteria and other microorganisms, here indicated by different colors. After total DNA has been extracted, the composition of the community is determined by amplifying and sequencing the 16S ribosomal RNA (rRNA) gene. Highly similar sequences are grouped into operational taxonomic Units (OTUs), which are labeled by comparison with databases of recognized organisms. OTUs can then be analyzed in terms of presence/absence, abundance, or phylogenetic diversity. In order to determine biomolecular and metabolic functions present in the community, the total metagenomic DNA may be sequenced and compared with function-oriented databases. Alternatively, sequenced community DNA can be compared with reference genomes. This allows identification of microbial sequence variants and polymorphisms and provides an alternative means of detecting the presence and abundance of specific organisms (adapted with permission from [18]). *KEGG* Kyoto Encyclopedia of Genes and Genomes, *BLAST* Basic Local Alignment Search Tool, *SNPs* single-nucleotide polymorphism(s)

The study of the human microbiome has been facilitated by technological advances in performing culture-independent analyses and has yielded remarkable insights into the complex diversity of the human microbiome [14]. While characterizing the microbial phylogenetic composition from a given body site describes only the archaeal and bacterial portion of the microbiome, metagenomic approaches identify all genomes existing in an environment, including bacteria, archaea, viruses, and eukaryotic microbes. Microbial phylogenetic and taxonomic applications are used to identify the microbiota composition using sequencing of the 16S ribosomal-RNA (rRNA)-encoding gene, followed by comparison to known bacterial sequence databases [15]. This method has its limitations, as it only provides insights into the taxonomic composition of the microbial community and fails to resolve a substantial fraction of the diversity existing in a community [16]. In other words, this approach allows the rapid determination of species occurrence and abundance in an efficient way for numerous samples simultaneously; however, it neglects the fact that identical species found in two different microbiomes might vary significantly in their functional capabilities [6]. To overcome these limitations, metagenomic analysis is used as an alternative approach to study the uncultured microbiota by sequencing all microbial DNA in a complex community [6, 16]. It has the additional advantage of assessing the genetic information of the microbial population and provides insights into the biological functions encoded in the microbial genome [16]. Other methodologies have also been designed to analyze the microbial transcriptome, proteome, and metabolome; they provide additional information at successive levels of microbial physiology [17]. Figure 2 summarizes some of the methodologies used to study the microbiome [18].

### The gut microbiome

The human gut is sterile at birth [19]. Colonization with a wide variety of microbes starts at birth originating from the mother’s vaginal and fecal microbiota, as well as from other environmental microbes encountered in the first days of life [19]. The adult human gut harbors a complex community of >100 trillion microbial cells, and >1000 different bacterial species constitute the gut microbiota [20]. It is estimated that this metabolically active endogenous “organ” is equivalent to 1–2 kg of body weight and is a reservoir of >1 g of endotoxin [21]. The gut microbiota composition varies greatly between individuals, with each individual harboring a unique collection of bacterial species, which is highly stable over time, and with the most abundant bacterial phyla found in the healthy human gut being the Gram-negative Bacteroidetes and the Gram-positive low-GC Firmicutes [22]. Many nutrients in the diet are digested by human enzymes and absorbed in the small intestine. However, the gut microbiota has a central role in the metabolism of dietary fibers, which are not degraded by human enzymes. In addition to its role in food digestion, the gut microbiota plays a role in stimulation of the immune system, maintenance of intestinal epithelium homeostasis, synthesis of vitamins (B and K), enhancement of gastrointestinal tract motility and function, nutrient absorption, inhibition of pathogens by creating colonization resistance, metabolism of plant-derived compounds/drugs, and production of short-chain fatty acids (SCFAs) and polyamines [22].

### Intestinal microbial dysbiosis and disease

Changes in composition and structure of the human microbiota, also known as dysbiosis, may predispose individuals to different disease conditions and explain why some people are more susceptible or resistant to certain diseases [23]. Alterations in the microbiota can result from exposure to various environmental factors, including diet, toxins, drugs, and pathogens [10]. Multiple studies have described relationships between gut microbial communities and disease states. Although these relationships are not cause-and-effect relationships, it is clear that the microbiome is an important contributor in many disease states, a factor that has been previously overlooked. In fact, changes in the microbiome are increasingly linked to the development of noncommunicable disease (NCD). Those include obesity [24], cancer [25, 26], diabetes [27, 28], inflammatory bowel disease (IBD) [29, 30], asthma [31], cardiovascular disease (CVD) [32, 33], kidney disease [21], and others. Therefore, understanding the interface between microbes and NCD may help uncover disease etiologies and pathogenesis. This may be achieved by identifying novel microbial causes or inflammatory intermediates that may be used as diagnostic and therapeutic targets for prediction, prevention, and treatment of common diseases. Deciphering the possible interindividual variations in microbial contents of the different body regions, and identifying changes in the human microbiota during onset or progression of various diseases is expected to leverage the application of microbiota-driven personalized medicine. While extensive research describing the role of the microbiome in obesity, IBD, cancer, and diabetes have been reported, only relatively recently have a few studies described the role of the microbiome in kidney disease [21, 34]. In this review, we address the role of dysbiosis in kidney disease, with a special focus on the role of the gut microbiome in chronic kidney disease (CKD).

### Chronic kidney disease

CKD is a global health issue associated with loss of kidney function, CVD, infectious diseases, and premature death [35]. This lethal synergy between CKD and CVD and the increased awareness of the limitations of current treatment options has prompted the nephrology and research communities to explore alternative therapeutics to improve outcomes. Loss of kidney function in CKD results in major alterations in the blood concentration of numerous molecules [36]. In particular, substances that would normally be excreted or metabolized by the kidney accumulate as renal function declines, resulting in increased blood concentrations [36]. These uremic retention molecules (URM) constitute a long and ever-expanding list of substances that upon accumulation substantially contribute to the syndrome of uremia [36]. URM are classified according to their origin: endogenous (mammalian metabolism), microbial, or exogenous (such as diet) [37, 38]. Although the majority of URMs originate endogenously from mammalian metabolism—like dimethylarginines, homocysteine, and oxalate—it is increasingly recognized that intestinal microbial metabolism also contributes to the generation of numerous URMs.

### CKD and the gut microbiome

Understanding the role of human gut microbiota in the progression of CKD requires a clear comprehension of the microbiota composition, dynamics, and stability within a patient. Gut microbes produce compounds that are normally excreted by the kidneys but can be considered as potential URM [39]. The principal role of the colon is to absorb salt and water and to provide a mechanism for orderly disposal of waste products of digestion. Moreover, the colon is responsible for salvage of energy and possibly nitrogen from carbohydrates and proteins that are not digested in the upper gastrointestinal tract. This is achieved through the metabolism of anaerobic bacteria, a process known as fermentation [39]. Fermentation of the amino acids tyrosine (obtained usually from consuming turkey, chicken, beef, brown rice, nuts, fish, milk, eggs, cheese, fruit, and vegetables) and tryptophan (e.g., from beef, poultry, pork, fish, milk, yogurt, eggs, cheese, and soy products) by intestinal microbiota generates p-cresol and indole, respectively. After absorption, these compounds are further metabolized in the liver to generate p-cresyl sulfate and p-indoxyl sulfate. Indoxyl sulfate and p-cresyl sulfate circulate in equilibrium between a free solute fraction and a fraction bound to serum proteins. The best characterized binding site is albumin, for which indoxyl sulfate and p-cresyl sulfate are competitive binding inhibitors [40]. These toxins are eliminated mainly by tubular secretion in the kidneys and, therefore, are considered to be uremic toxins, with increased levels indicative of renal impairment and advancing CKD [41].

Dysbiosis in CKD patients may contribute to increased uremic toxin levels that in turn contribute to CKD progression. In a prospective, observational study of 268 patients with CKD, Wu and colleagues found the baseline concentration of indoxyl sulfate to be predictive of CKD progression [42]. Meijers and colleagues measured p-cresol levels in 499 patients with mild-to-moderate CKD and showed that p-cresol sulfate levels increased with decreasing estimated glomerular filtration rate (GFR) [43]. Likewise, an elevated p-cresol concentration was associated with increased risk of death in end-stage renal disease (ESRD) patients treated with maintenance hemodialysis [44]. Trimethylamine N-oxide (TMAO) is another uremic toxin produced by the gut microbiome, and its role in CKD has also been examined [45]. In a large cohort of CKD patients, Tang and colleagues found elevated TMAO concentrations in patients with CKD. These elevated concentrations were associated with a 70 % higher risk for all-cause mortality, even after adjusting for traditional risk factors and C-reactive protein [46].

It is worth noting that the interaction between the gut microbiota and CKD is not unidirectional. CKD also affects the structure of the gut microbiota and contributes to dysbiosis. In healthy individuals, gut microbiota are classified into different enterotypes based on the abundance of specific bacterial groups, which are dominated by *Bacteroides*, *Prevotella*, or *Ruminococcus* [47]; these enterotypes are strongly associated with long-term diets, particularly the levels of proteins and animal fat (*Bacteroides*) versus carbohydrates (*Prevotella*) [48]. However, the intestinal microbiota in patients with CKD is altered, with lower numbers of *Lactobacillaceae* and *Prevotellaceae* families (both are considered normal colonic microbiota) and 100 times higher *Enterobacteria* and *Enterococci* species (which are normally present in lower proportions) [49].

**Fig. 3:**
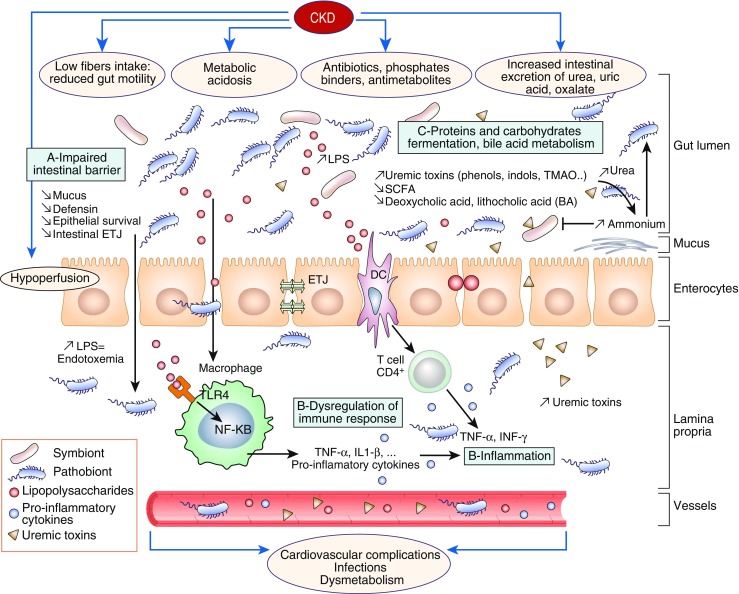
Dysbiosis and chronic kidney disease (CKD). CKD impairs the balance between symbionts and pathobionts in a way that favors pathobiont overgrowth. Consequences are as follows: **a** Impairment of the intestinal barrier by disrupting the colonic epithelial tight junction (ETJ) and decreasing epithelial survival. An increase in loss of integrity in intestinal permeability allows translocation of bacteria and lipopolysaccharide (LPS). **b** Dysregulation of immune response and inflammation. LPS could activate innate immune cells through toll-like receptor 4 (TLR4)-dependent and nuclear factor kappa B (NF-κB) pathways. Pathobionts stimulate dendritic cells (DCs) that activate a Th17/Th1 T-cell response and enhance production of inflammatory cytokines. **c** Modification of carbohydrates, protein, and bile acid (BA) fermentation. Proteins are fermented by intestinal pathobionts, which are then converted preferentially into indoxyl-sulfate (IS), p-cresyl sulfate (PCS), and trimethylamine n-oxide (TMAO). The reduction in symbionts, specifically Bifidobacterium, induces a decrease in short-chain fatty acids (SCFAs). Dysbiosis modifies BA levels and composition. *INF-γ* interferon γ, *IL-1* interleukin-1, *TNF-α* tumor necrosis factor-α. (Adapted with permission from [57])

Kidney disease is associated with decreased consumption of dietary fibers [50], frequent use of antibiotics [51], slow colonic transit, metabolic acidosis, volume overload with intestinal wall congestion, intestinal wall edema, and oral iron intake [52–54]. These factors are also associated with microbial dysbiosis and higher numbers of pathogenic microbes in the gut. Many of these factors affect intestinal tight junctions and result in increased intestinal permeability and translocation of bacterial products across the intestinal barrier that will, in turn, trigger an immune response. The latter could explain the systemic inflammation that is associated with and contributes to worsening CKD and CVD [55]. Another possible mechanism of microbial dysbiosis in patients with CKD results from increased gastrointestinal urea secretion [56]. Urea is hydrolyzed by gut microbes, resulting in the formation of large quantities of ammonia, which affects the growth of commensal bacteria and causes imbalance in the gut microbiota [56]. Figure 3 summarizes mechanisms and pathways of dysbiosis in patients with CKD [57].

Therefore, targeting the large intestine and understanding the composition of the gut microbial communities might be a promising adjuvant approach to tackle the high morbidity and mortality in patients with CKD.

### Hypertension and the gut microbiome

About 70 million Americans (29 %) have high blood pressure, which accounts for one out of three adults [58]. It is estimated that >3 % of children have hypertension [59]; this number is much higher in obese children, since the prevalence of hypertension rises progressively with increases in body mass index (BMI) percentile from ≤5th (2 %) to ≥95th (11 %) [60]. The relationship between kidney disease and hypertension is bidirectional. The microbiota of a small cohort of hypertensive patients was described as less rich and diverse than that of controls [61]. Pluznick et al. reported that major components of the olfactory signaling pathway are present in the kidney; Olfr78 is an olfactory receptor expressed in the renal juxtaglomerular apparatus, where it mediates renin secretion in response to SCFAs. Those fatty acids are end products of fermentation by the gut microbiota and are absorbed into the circulation. Treatment with antibiotics reduced the biomass of the gut microbiota and elevated blood pressure in *Olfr78* knockout mice [62]. Another possible link between the gut microbiota and hypertension comes from the intestinal microbiota metabolism of choline and phosphatidylcholine, which produces trimethylamine (TMA), which is further metabolized to a proatherogenic species: TMAO. Koeth and colleagues demonstrated that metabolism by intestinal microbiota of dietary L-carnitine, a TMA abundant in red meat, produces TMAO and accelerates atherosclerosis in mice [63].

### Gut microbiome and other renal conditions

Recently, multiple studies have examined the relationship between different kidney disease entities and the gut microbiome. De Angelis and colleagues examined the gut microbiome in progressor vs nonprogressor immunoglobulin A (IgA) nephropathy (IgAN) patients and compared them with healthy controls [64]. They showed that some traits of the gut microbiota and levels of urinary metabolites (free amino acids and organic volatile compounds) vary significantly between the progressor and nonprogressor groups [64]. It was hypothesized that the increased free amino acids in the serum due to IgAN pathology is possibly related to the decreased absorption of gastrointestinal proteins, which allowed for increased microbial proteolysis, altered microbiota, and contributed to elevated fecal p-cresol levels. Recent data indicate that intestinal microbiota can modify acute kidney injury (AKI) also. One possible mechanism is via the renoprotective action of SCFAs against ischaemia–reperfusion in animal models; SCFAs (which have anti-inflammatory properties) are produced by the intestinal microbiota [65]. Therapy with three main SCFAs (acetate, propionate, and butyrate) improved renal dysfunction caused by injury and was associated with reductions in the levels of reactive oxygen species, inflammation, infiltrating immune cells, and apoptotic cells in the injured kidneys; an increase in proliferation of kidney epithelial cells; and modulation of DNA methylation status. Another possible mechanism by which the microbiota affect AKI outcome is related to the hygiene hypothesis [66]; Jang and colleagues reported that germ-free animals when subjected to ischemia-induced AKI have significantly worse structural/functional renal injuries and inflammation compared with control mice. This may be due to a T-helper 1 type response similar to that seen in autoimmune disease. Furthermore, the microbiota may have a wider influence and role in autoimmune kidney disease through its immunomodulatory effects, recognized by its influence on polarization of T-cell subsets and natural killer cells [67]. Abnormalities in the immune system can also induce kidney damage, either by provoking an autoimmune phenomenon or induction of molecular mimicry. *Staphylococcus aureus* may be a direct pathogenetic factor in granulomatosis with polyangiitis (former Wegener’s granulomatosis) [68]. Chronic bacterial colonization or chronic infections of the upper respiratory tract have been suspected to be a trigger of IgA vasculitis and IgAN [69]. Microbial antigens may play a role in membranous nephropathy and lupus nephritis, as suggested by the abundance of *Helicobacter pylori* antigen deposition in renal biopsy specimens and evidence of infection in blood [70]. Undergoing kidney transplantation and the use of posttransplant medications has a major impact on the microbiota composition as well [71]. On the other hand, differences in the gut microbiota may affect medication bioavailability and dosing to achieve therapeutic levels; this may help further explain interindividual differences in tacrolimus dosing [72] and how antibiotic intake may increase the bioavailability of amlodipine and possibly change its therapeutic potency by suppressing gut microbial metabolic activities [73].

### Restoring the balance: current and potential interventions

These interventions can be classified as being designed to lower uremic toxin production by restricting intake of uremic toxin precursors (lower protein intake), by restoring a more balanced gut microbiome (food supplements), and by interventions aiming to enhance the disposal of these toxins (adsorptive therapies).

#### Food supplements

##### Prebiotics

Prebiotics is a general term to refer to nondigestible (by the host) food ingredients that induce the growth or activity of microorganisms (e.g., bacteria and fungi) that contribute to the well-being of their host. Inulin, fructo-oligosaccharides, galacto-oligosaccharides, soya-oligosaccharides, xylo-oligosaccharides, and pyrodextrins are among known prebiotics. There are limited studies examining their effect in CKD patients. In a randomized, controlled, single-blind clinical trial with a crossover design, Bliss and colleagues studied the effect of gum arabic (acacia gum) fiber supplementation (50 g/day) in 16 CKD patients consuming a low-protein diet [74]. Four weeks after receiving gum arabic fiber, patients had increased fecal nitrogen excretion and lower serum urea nitrogen concentration compared with the placebo group. Only one small study of three pediatric patients with ESRD examined the efficacy of acacia gum (1 g/kg per day in divided doses) and reported improved quality of life [75]. Another randomized controlled trial examined the effect of increasing dietary fiber on plasma levels of colon-derived solutes in 56 hemodialysis patients [76]. After 6 weeks, patients on increasing dietary fiber had significantly reduced unbound, free plasma levels of indoxyl sulfate, while the reduction of p-cresol sulfate levels did not achieve significance. The authors concluded that increasing dietary fiber in hemodialysis patients might reduce plasma levels of the colon-derived solutes indoxyl sulfate and possibly p-cresol sulfate without the need to intensify dialysis treatments.

##### Probiotics

Probiotics are living microorganisms that, when administered in adequate amounts, confer a health benefit on the host. Studies examining the effects of probiotics in CKD patients using serum tumor necrosis factor alpha (TNF-α), interleukin (IL)-5 and -6, and endotoxin show conflicting results. In a randomized, double-blind, placebo-controlled trial examining the effect of probiotics on serum cytokine and endotoxin levels of patients on peritoneal dialysis, patients who received probiotics had lower proinflammatory cytokines and endotoxin levels while levels of serum IL-10 significantly increased [77]. In a recent study, Yacoub et al. analyzed National Health and Nutrition Survey (NHANES) data to examine the association of yogurt/probiotic with kidney parameters. Frequent yogurt and/or probiotics use was associated with decreased risk of proteinuric kidney disease [78].

##### Synbiotics

Synbiotics refer to nutritional supplements combining probiotics and prebiotics in a form of synergism. In a randomized, placebo-controlled trial, Guida and colleagues studied the effect of a 4-week synbiotic treatment on plasma p-cresol levels in 30 patients with stage 3–4 CKD [79]. The authors found the group on synbiotics to have lower total plasma p-cresol concentrations and suggested that because high plasma concentrations of p-cresol in early phases of CKD are predictive of progression to ESRD, synbiotics deserve attention as possible tools to delay CKD progression. Others have reported similar beneficial effects of synbiotic and low-protein treatment on CKD progression [80]. In a recent randomized trial, Rossi et al. evaluated the effect of synbiotics therapy on gut microbiota and serum concentrations of indoxyl sulfate and p-cresyl sulfate in predialysis CKD [81]. Synbiotic therapy did not significantly reduce serum indoxyl sulfate levels but reduced levels of p-cresyl sulfate and favorably altered the stool microbiome, particularly with enrichment of *Bifidobacterium* and depletion of *Ruminococcaceae* [81].

#### Adsorbent therapies

AST-120 is an orally ingested intestinal spherical carbon adsorbent consisting of porous carbon particles of 0.2–0.4 mm in diameter and is insoluble in water and common organic solvents. It adsorbs indole, the precursor of indoxyl sulfate derived from the metabolism of tryptophan by bacteria within the gastrointestinal tract and therefore is used to attenuate indoxyl sulfate accumulation in patients with CKD [82]. AST-120 has been available in Japan since 1991. In prospective trials and retrospective analyses, AST-120 has been shown to prolong the time to initiation of hemodialysis [83] and slow the decline in GFR and the increase in serum creatinine [84]. In an initial randomized, double-blind, placebo-controlled trial in the United States, AST-120 was associated with a significant dose-dependent reduction in serum indoxyl sulfate levels and a decrease in uremia-related malaise [85]. The Evaluating Prevention of Progression in CKD (EPPIC) trials, two double-blind, placebo-controlled trials undertaken in North America/Latin America and Europe, evaluated the efficacy of AST-120 for preventing progression of CKD in 2035 adults with moderate to severe disease. Patients we randomized to receive either placebo or AST-120 (9 g/d). The study found the time to primary end point (a composite of dialysis initiation, kidney transplantation, and serum creatinine doubling) to be similar between the AST-120 and placebo groups in both trials. The authors concluded that the benefit of adding AST-120 to standard therapy in patients with moderate to severe CKD is not supported by their study data [86].

#### Fecal microbiota transplantation (FMT)

FMT is becoming increasingly accepted as an effective and safe intervention in patients with recurrent *Clostridium difficile* infection, aiming at the restoration of a disrupted microbiome [87]. While no large studies examining the effect of fecal transplantation on restoring the gut microbiome in patients with CKD are available, a case showing successful eradication of a pathogenic organism in a patient with ESRD has been reported [88]. Whether healthy microbial transfer “in a pill” will be part of our future management of patients with CKD and dysbiosis remains to be seen. Until then, further studies are needed to describe what a healthy microbiome is in a patient with CKD and what hard clinical outcomes can be achieved from manipulating the microbiome.

## Conclusion

The relationship between the human microbiome and kidney disease is bidirectional. Recent studies have described how kidney disease contributes to dysbiosis and how dysbiosis contributes to progression of kidney disease. Clinicians must be aware of the potential, unintended effects of treatments that may alter the gut microbiome, exercise self-discipline, and weigh risks and benefits when prescribing prophylactic antibiotics to patients with recurrent urinary tract infections, vesicourethral reflux, and other infections. There is a pressing need for more studies that characterize the microbiome profile in children with CKD and explore the relationship between different pediatric kidney disease parameters and the microbiome of the growing child. This is not only needed to establish relationships by association but to examine the interaction between certain early-life microbial and antibiotic exposure on the pathogenesis of kidney disease. Multiple promising interventions have been described to restore a more balanced microbiome and possibly slow the progression of CKD; such interventions need to be further examined in large controlled trials before they can become part of our mainstream management.
